# Asthma Action Plans: An International Review Focused on the Pediatric Population

**DOI:** 10.3389/fped.2022.874935

**Published:** 2022-04-26

**Authors:** Francesco Pegoraro, Marzio Masini, Mattia Giovannini, Simona Barni, Francesca Mori, George du Toit, Irene Bartha, Enrico Lombardi

**Affiliations:** ^1^Department of Health Sciences, University of Florence, Florence, Italy; ^2^Allergy Unit, Department of Pediatrics, Meyer Children’s University Hospital, Florence, Italy; ^3^Pediatric Allergy Group, Department of Women and Children’s Health, School of Life Course Sciences, King’s College London, London, United Kingdom; ^4^Children’s Allergy Service, Evelina London Children’s Hospital, Guy’s and St Thomas’ NHS Foundation Trust, London, United Kingdom; ^5^Peter Gorer Department of Immunobiology, School of Immunology & Microbial Sciences, King’s College London, London, United Kingdom; ^6^Pulmonary Unit, Department of Pediatrics, Meyer Children’s University Hospital, Florence, Italy

**Keywords:** asthma action plans, international, children, education, treatment

## Introduction

Asthma is a chronic respiratory disease that affects more than 339 million people worldwide (1). It is the most common chronic respiratory disease in children, and its prevalence varies widely between nations (2, 3), ranging from 2.6% in Albanian children to 26.5% in Singaporean teenagers (4). Management of asthma and especially of its exacerbations is a main concern in children since asthma activity may contribute to causing bronchial remodeling in the future (5–7). Several longitudinal studies have shown that uncontrolled asthma in childhood is associated with a permanent decrease in lung function well into adulthood. Furthermore, lower values of Forced Expiratory Volume in one second (FEV1) in childhood asthma are directly associated with an increased risk of developing Chronic Obstructive Pulmonary Disease (COPD) in adulthood (5, 8, 9). Thus, optimal asthma control in children is of paramount importance for preventing life-long debilitating respiratory conditions.

Asthma action plans (AAPs) are a set of written instructions for the patients to follow in their day-to-day management, as their asthma worsens or in case of exacerbation. These plans are usually personalized, according to patients’ clinical characteristics, controller treatments, common triggers, and other factors. AAPs are supposed to facilitate the recognition of exacerbations or declining lung function, and also contain instructions on how to manage or modify, to an extent, the long-term controller therapy in case of worsening clinical manifestations.

Asthma action plans should be designed to be clear and practical sets of algorithmic instructions, with eye-catching and distinct features to facilitate patient understanding and compliance: they usually employ a three-zone, “traffic light” scheme, ranging from a green zone (which corresponds to an absence of signs and symptoms, with no need to modify therapy) to a red zone (in which patients are instructed on how to recognize clinical manifestations of generally acute and severe loss of control and to seek immediate medical attention together with the administration of rescue therapy). A yellow zone is usually present as well, corresponding to a progressively declining control and worsening of clinical manifestations, with instructions on how to escalate controller therapy accordingly. This last point seems the more complex and delicate one, and to date, guidelines struggle to give clear-cut and practical indications on how to structure it (10–12). A recent work from Kouri et al. (13) gave a valuable tool on how to implement action plans, especially regarding the yellow zone in the adult asthmatic population. Unfortunately, the same attention has not been paid to pediatric AAPs, even though a suboptimal control of asthma in childhood puts patients at risk of long-term complications, as mentioned earlier (5, 8, 9).

Pediatric AAPs pose peculiar problems in drafting, given the characteristics of the population to whom they are directed: e.g., different response to therapy, suboptimal compliance especially in adolescents, and the mandatory involvement and education of parents or legal guardians, as well as other figures as teachers, are all variables that should be taken into account in a pediatric AAPs but are not constantly considered, and when they are considered, they are implemented in vastly different ways.

Several studies have shown the benefits of providing action plans to asthma patients, both in the adult (14, 15) and pediatric population (16), resulting in reduced frequency of exacerbations, fewer visits to the Emergency Department, and an overall improvement of the quality of life. Following this, international asthma recommendations suggest that all asthmatic patients receive a written action plan (10–12). The importance of these plans got even greater during the Severe Acute Respiratory Syndrome Coronavirus 2 (SARS-CoV-2) pandemic, considering the possibility of an exacerbation during a national lockdown and the potential difficulties in contacting doctors. However, an overwhelming number of patients are not provided with an action plan (17–21).

This study aims to review and compare the characteristics and differences between action plans currently proposed by English-speaking societies and, most importantly, their applicability and limitations in the pediatric population (<18-year-old).

## Materials and Methods

### Asthma Action Plan Collection

We collected AAPs recommended by the found English-speaking organizations, i.e., from the United Kingdom (Asthma United Kingdom), Ireland (Asthma Society of Ireland), the United States (American Academy of Allergy Asthma & Immunology, Asthma and Allergy Foundation of America, American Lung Association, National Institutes of Health), Canada (Asthma Canada), New Zealand (Asthma Respiratory Foundation New Zealand), Australia (National Asthma Council Australia), and South Africa (Allergy Foundation South Africa) and they were evaluated. We initially screened for pediatric AAPs and then included adult AAPs when a society did not provide a plan specifically addressed to children. When both adult and pediatric plans were available from the same society, we included both to allow a comparison between them. AAPs designed by single centers (hospitals and clinics) or subnational organizations were not included.

### Asthma Action Plan Analysis

A literature search on the main interfaces (PubMed, Embase, Cochrane) and the websites of English-speaking organizations was performed from January 2010 to October 2021, to identify updated recommendations on how to design asthma action plans (11–13, 15, 22–24) and to collect all the essential features/items for effective AAPs. Each AAP was then evaluated to define whether it conformed to such features. During the examination, we identified items that we had not considered; when such items were deemed potentially helpful, they were included in the analysis. Finally, 27 items were deemed worthy inclusion: their detailed description is reported in Supplementary Table 1.

## Results

A total of 16 plans provided by 10 organizations were included in the analysis (Supplementary Table 2). Five organizations (50%) provided specific plans for adult and pediatric patients, one (10%) additional plan included information that could fit both adults and children, whereas four plans (40%) lacked any reference to children’s care.

The detailed summary of our analysis is reported in Supplementary Table 3. Among plans, written information for the parent or guardian was included in four (40%). All but one plan (90%) pointed out an emergency contact, while a phone or web address for asthma education was available in four (40%). A space for a photograph was added to one plan only (10%). All plans included information on the date of the last review, the referring clinician who revised it, and the patient’s personal best peak flow. Potential factors exacerbating the patient’s asthma, such as environmental triggers or physical exercise, were detailed in six (60%) and five (50%) plans, respectively.

A section to report additional, personalized clinical characteristics was included in five plans (50%). All plans followed a severity-based three-step approach to asthma exacerbation management—only the Asthma and Respiratory Foundation of New Zealand designed an additional four-step adult plan.

A detailed description of the sequential management steps was provided in all cases, including a list of asthma medications for every day and exacerbation use. Four plans (40%) included an additional description of asthma medication colors for better recognition in emergency settings, whereas two (20%) included an explanation of asthma medication functioning. The need for a spacer for therapy administration and information on oral glucocorticoids use were both detailed in six plans (60%). Additional detailed information on how to administer therapy was reported in two plans (20%). Only one plan (10%) included an explicit authorization to administer medications in case of emergency. Three plans (30%) included extra information on asthma first-aid procedures, whereas two plans (20%) focused on recovery and follow-up after an exacerbation. Additional information on comorbidities and asthma-independent therapies were included in three plans (30%). No plan mentioned anaphylaxis potentially associated with asthma exacerbation, nor its management.

## Discussion

It has been documented that providing asthmatic patients with a written action plan results in a better asthma control and reduced visits to the Emergency Department, both in adults and children (16, 24). However, out of all the action plans we examined, only five provided a specific action plan targeted toward children. This finding raises concerns since optimal pediatric asthma management is crucial for preventing a long-term permanent decline in lung function resulting from poor asthma control (8, 9, 25). Moreover, asthma in children and adults are considered two different entities worthy of different therapeutic approaches, as stated in the latest Global Initiative for Asthma (GINA) report (12), in which different indications are given even between different age ranges among pediatric patients.

We found substantial heterogeneity even among action plans that included a pediatric version, especially when considering aspects that might appear trivial in the adult setting but gain great importance when dealing with children. Specific instructions provided toward the caregiver on how to behave in the event of an exacerbation were provided in a varied way. The identification of medications by their color, instructions on how to administer therapy (including the use of a spacer), and the presence of comorbidities are of fundamental importance since signs and symptoms of asthma might have to be managed by people other than the patient, and especially by people who might be inexperienced in the treatment of asthma, like teachers and trainers. Unexpectedly, an important element such as the authorization to administer therapy in emergencies was mentioned in only one plan.

In addition to differences when dealing with the specific pediatric issues of asthma management, we noticed differences in the inclusion of steps that could be deemed crucial both in a pediatric (and in an adult setting), as the lack of explicit recommendation of the use of systemic corticosteroids in half of the plans, even though their use is recommended in both the GINA report and the CTS guidelines (12, 26). When looking specifically at “red zones”, only one out of all the plans mentioned the use of systemic corticosteroids.

It must be acknowledged that including corticosteroids in an action plan is not easy, considering the different ways of using it in terms of ways of administration (oral *versus* inhaled) and indications (rescue versus maintenance treatment). Likewise, other therapeutic approaches, such as ipratropium bromide and the maintenance and reliever therapy (MART), might find their place in AAPs in the future.

**FIGURE 1:**
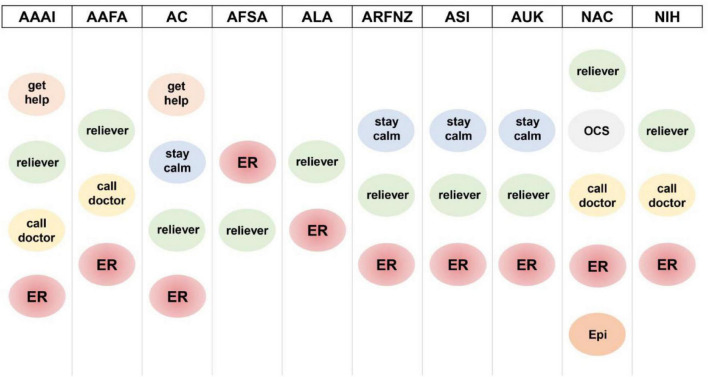
Instructions provided by the action plans in case of acute asthma exacerbation. *get help*: the plan recommends seeking assistance (either medical or not) during an exacerbation; *reliever*: the plan recommends administering the reliever drug; *call doctor*: the plan recommends contacting the family/emergency physician; *stay calm*: the plan recommends not panicking and following the instructions included in the plan; *ER*: the plan recommends reaching the nearest emergency room; *OCS*: the plan recommends taking oral corticosteroids; *Epi*: the plan recommend administering epinephrine if indicated. AAAI, American Academy of Allergy Asthma & Immunology; AAFA, Asthma and Allergy Foundation of America; AC, Asthma Canada; AFSA, Allergy Foundation South Africa; ALA, American Lung Association; ARFNZ, Asthma Respiratory Foundation New Zealand; ASI, Asthma Society of Ireland; AUK, Asthma United Kingdom; NAC, National Asthma Council Australia; NIH, National Institutes of Health.

We focused on the different recommended sequences of actions to enact in the event of a severe exacerbation, the escalation of therapy, and the need for assistance (Figure 1). As expected, the two most important actions to perform in case of severe exacerbation (i.e., “administer reliever” and “call an ambulance”) are always present—despite not always reported in the same order. However, other significant steps and recommendations are often not explicitly stated.

Only two of them explicitly advised the caregiver to get help, an important step to describe when instructing someone who might be inexperienced in administering medication in a potentially critical setting and whose attention might be monopolized by the distressed child and might not be able to call for an ambulance, another crucial step, indeed mentioned in all the action plans we examined.

We believe that instructing the child and the caregiver holds significant weight in both the medications to take and the behavior to adopt. Directions like “stay calm,” “sit straight,” and “take slow, steady breaths” can help tremendously in an asthma exacerbation. However, most of the examined documents lacked practical instructions on the actions to take in an acute loss of signs and symptoms control, only referencing the medications to take/administer.

Despite being a valuable tool in managing asthma in adults and children, we found that AAPs show significant variability and different degrees of comprehensiveness in their approach to the asthmatic patient. AAPs are not frequently adapted to the peculiarities of the pediatric patient, and those flaws are especially apparent when considering an emergency setting and the caregiver’s involvement. Providing caregivers with clear and easy-to-follow instructions is essential when dealing with emergency situations. Moreover, it is mandatory to ensure the completeness of AAPs, including other situations that might associate with asthma.

Overall, AAPs are effective tools for the management of patients with asthma. However, their heterogeneity will have to be addressed to improve AAP quality and effectiveness. Indeed, even if a certain grade of variability could be normal due to differences in several populations, the effectiveness of the management of asthma patients could improve with the standardization of the items included in AAPs. Moreover, it would be of the utmost importance for pediatric AAPs to be conceived with a 360-degrees view of the child in mind as a different and unique entity, worthy of a dedicated approach.

## Author Contributions

MG and EL conceptualized the work. FP, MM, and IB collected the data and drafted the manuscript. FP, MM, MG, SB, FM, GdT, IB, and EL analyzed the data. MG, SB, FM, GdT, and EL critically revised the manuscript. All authors approved the final version of the manuscript as submitted.

## Conflict of Interest

The authors declare that the research was conducted in the absence of any commercial or financial relationships that could be construed as a potential conflict of interest.

## Publisher’s Note

All claims expressed in this article are solely those of the authors and do not necessarily represent those of their affiliated organizations, or those of the publisher, the editors and the reviewers. Any product that may be evaluated in this article, or claim that may be made by its manufacturer, is not guaranteed or endorsed by the publisher.
