# Supplementary material for: Asthma Action Plans: An International Review Focused on the Pediatric Population
Source: Front Pediatr. 2022 Apr 26;10:874935. doi: 10.3389/fped.2022.874935 (PMC9113391; doi:10.3389/fped.2022.874935)
Supplement: Supplementary file 1 [file Table_1.pdf]

Table S1. Detailed description of the features included in the analysis of the action plans

Adult plan	There is a dedicated action plan for patients aged 16 and above
Pediatric plan	There is a dedicated action plan for patients aged 6-16
Adult and pediatric plan	There is a unique plan for adults and children
Information for parent/guardian	The plan provides a set of instructions that a parent or guardian (e.g., teacher) should follow in the event of an asthma attack
Emergency contact	The plan reports the phone numbers to contact in case of an asthma attack: alternative physician, asthma specialist, emergency room
Asthma education contact	The plan provides a phone number, an address and/or an e-mail of the asthma specialist responsible for the patient's follow-up
Doctor information	The plan reports contacts of the patient's family doctor/pediatrician
Photography	There is a photo ID of the patient
Authorization to administer medicines	A form authorizing the administration of drugs to by adults (other than the patients' parents) caring for the pediatric patient at the moment of exacerbation is included
Last plan review	The date of the last time the plan was reviewed, checked, and adjusted according to the patient's asthma severity, control, age, and weight is reported
Focus on triggers	The plan has a section describing the different triggers to be wary of to avoid the occurrence of exacerbations
Focus on exercise	The plan has a section describing how to behave when practicing sport/physical exercise and when/which medications to take if needed
Peak flow	The plan uses peak flow measurement to monitor asthma control
Additional tailored clinical information	The plan has a dedicated space to allow the inclusion of additional personalized information specific to the patient, not included in the pre-compiled sections
Severity classification	The plan present separate severity levels dictated by sign, symptoms and/or PEF measurement
List of asthma medications	The plan reports the list of specific asthma medications used by the patient
Colors of asthma medications	The plan reports the colors of specific asthma medications used by the patient
Comorbidities/other treatments	The plan mentions conditions other than asthma that can interfere with asthma management or conditions to be wary of while caring for the patient experiencing an asthma attack
Management of asthma attacks	The plan provides practical instructions on how to behave in the event of an asthma exacerbation (other than drug administration)
Reference to anaphylaxis and its management	The plan provides instructions on how to differentiate asthma attacks from anaphylactic reactions and how to address a suspected anaphylactic reaction
Information on asthma first-aid	There is a section explaining the primary care in case of an asthma attack
Information on asthma medication functioning	The plan describes the mechanism of action of medications and their different purposes and effects
Information on how to administer therapy	The plan describes the methods used to administer (inhaler) therapy in the most effective way
Information on recovery and follow-up	The plan describes the steps to take after the exacerbation has been managed and asthma control has been re-established
Mentions OCS use	The plans describe when to use OCS and the dosage to take

Precompiled instructions/steps description	Instructions on how to recognize a worsening control and how to escalate therapy are included
Spacer mentioned	The plan encourages the use of a spacer when taking medications via an inhaler

Abbreviations used in the table. PEF: peak expiratory flow; OCS: oral corticosteroids
