# Supplementary material for: Asthma Action Plans: An International Review Focused on the Pediatric Population
Source: Front Pediatr. 2022 Apr 26;10:874935. doi: 10.3389/fped.2022.874935 (PMC9113391; doi:10.3389/fped.2022.874935)
Supplement: Supplementary file 2 [file Table_2.pdf]

**Table S2.** Included asthma action plans

| Scientific society | Plan | URL |
|---|---|---|
| American Academy of Allergy Asthma & Immunology (AAAI) | AAAI_2011 | https://www.aaaai.org/Aaaai/media/MediaLibrary/PDF%20Documents/Libraries/16-asthma-action-plan-v10_hires.pdf |
| Asthma and Allergy Foundation of America (AAFA) | AAFA_2018_adult | https://secure.aafa.org/np/clients/aafa/product.jsp?product=21& |
| | AAFA_2018_child | https://secure.aafa.org/np/clients/aafa/product.jsp?product=12& |
| Asthma Canada (AC) | AC_adult | https://asthma.ca/wp-content/uploads/2020/06/Asthma-Action-Plan_optimized.pdf |
| | AC_child | https://asthma.ca/wp-content/uploads/2019/08/Kids-Action-Plan-FINAL.pdf |
| Allergy Foundation South Africa (AFSA) | AFSA_2017 | https://www.allergyfoundation.co.za/wp-content/uploads/2016/11/38-asthma-action-plan.pdf |
| American Lung Association (ALA) | ALA_adult | https://www.lung.org/getmedia/1bb7284c-4b2b-45da-b54d-c24012207957/asthma-action-plan.pdf.pdf |
| | ALA_child | https://www.lung.org/getmedia/aa8ce6f5-667e-4726-b4ab-8ac8d5d448e4/fy20-ala-asthma-action-plan_home_school.pdf |
| Asthma Respiratory Foundation New Zealand (ARFNZ) | ARFNZ_3steps | https://www.asthmafoundation.org.nz/assets/documents/ARFNZ-3-Stage-Asthma-Action-Plan-2021-06-interactive.pdf |
| | ARFNZ_4steps | https://www.asthmafoundation.org.nz/assets/documents/ARFNZ-4-Stage-Asthma-Action-Plan-2021-06-interactive.pdf |
| | ARFNZ_child | https://www.asthmafoundation.org.nz/assets/documents/172460-Child-Asthma-Plan_ASTH10_web.pdf |
| Asthma Society of Ireland (ASI) | ASI_2018 | https://www.asthma.ie/sites/default/files/files/document_bank/2018/Nov/ASI%20-%20Asthma%20Action%20Plan%202018.pdf |
| Asthma United Kingdom (AUK) | AUK_2021_adult | https://www.asthma.org.uk/ac76e7a2/globalassets/health-advice/resources/adults/asthma-action-plan-adult-2021.pdf |
| | AUK_2021_child | https://www.asthma.org.uk/e75bf921/globalassets/health-advice/resources/children/my-asthma-plan-2021-v5-multi-media-live.pdf |
| National Asthma Council Australia (NAC) | NAC_2015 | http://s3-ap-southeast-2.amazonaws.com/nationalasthma/resources/341-NAC-Written-Asthma-Action-Plan-2015_Colour.pdf |
| National Institutes of Health (NIH) | NIH_2017 | https://www.nhlbi.nih.gov/files/docs/public/lung/asthma_actplan.pdf |
