# Supplementary material for: Asthma Action Plans: An International Review Focused on the Pediatric Population
Source: Front Pediatr. 2022 Apr 26;10:874935. doi: 10.3389/fped.2022.874935 (PMC9113391; doi:10.3389/fped.2022.874935)
Supplement: Supplementary file 3 [file Table_3.pdf]

Table S3. Analysis of asthma action plans

	AAAI	AAFA	AC	AFSA	ALA	ARFNZ	ASI	AUK	NAC	NIH
Adult plan	X	X	X	X	X	X		X	X	X
Pediatric plan		X	X		X	X		X		
Adult and pediatric plan							X			
Information for parent/guardian		X	X		X	X				
Emergency contact	X	X	X		X	X	X	X	X	X
Asthma education contact			X			X	X	X		
Doctor information	X	X	X	X	X	X	X	X	X	X
Picture		X								
Authorization to administer medicines					X					
Last plan review	X	X	X	X	X	X	X	X	X	X
Focus on triggers		X	X		X		X	X		X
Focus on exercise		X			X	X	X			X
Peak expiratory flow	X	X	X	X	X	X	X	X	X	X
Personalized clinical information	X	X	X					X	X	
Severity classification	X	X	X	X	X	X	X	X	X	X
List of asthma medications	X	X	X	X	X	X	X	X	X	X
Colors of asthma medications			X			X	X	X		
Comorbidities/other treatments			X				X		X	
Management of asthma attacks	X	X	X	X	X	X	X	X	X	X
Reference to anaphylaxis and its management										
Information on asthma first-aid						X	X	X		
Information on asthma medication functioning						X			X	
Information on how to administer therapy				X		X				
Information on recovery and follow-up				X				X		
Information on oral corticosteroids use				X		X	X	X	X	X
Instructions/steps description	X	X	X	X	X	X	X	X	X	X
Information on spacer			X	X		X	X	X	X	

Abbreviations used in the table. AAAI: American Academy of Allergy Asthma & Immunology; AAFA: Asthma and Allergy Foundation of America; AC: Asthma Canada; AFSA: Allergy Foundation South Africa; ALA: American Lung Association; ARFNZ: Asthma Respiratory Foundation New Zealand; ASI: Asthma Society of Ireland; AUK: Asthma United Kingdom; NAC: National Asthma Council Australia; NIH: National Institutes of Health
